# The impact of patient-reported outcome (PRO) data from clinical trials: a systematic review and critical analysis

**DOI:** 10.1186/s12955-019-1220-z

**Published:** 2019-10-16

**Authors:** Samantha Cruz Rivera, Derek G. Kyte, Olalekan Lee Aiyegbusi, Anita L. Slade, Christel McMullan, Melanie J. Calvert

**Affiliations:** 1 Centre for Patient Reported Outcomes Research, Institute of Applied Health Research, College of Medical and Dental Sciences, University of Birmingham, Birmingham, UK; 2 NIHR Birmingham Biomedical Research Centre, NIHR Surgical Reconstruction and Microbiology Research Centre University Hospitals Birmingham NHS Foundation Trust and University of Birmingham, Birmingham, UK

**Keywords:** Patient-reported outcomes, Quality of life, Impact, REF case studies, Clinical trials

## Abstract

**Background:**

Patient-reported outcomes (PROs) are commonly collected in clinical trials and should provide impactful evidence on the effect of interventions on patient symptoms and quality of life. However, it is unclear how PRO impact is currently realised in practice. In addition, the different types of impact associated with PRO trial results, their barriers and facilitators, and appropriate impact metrics are not well defined. Therefore, our objectives were: i) to determine the range of potential impacts from PRO clinical trial data, ii) identify potential PRO impact metrics and iii) identify barriers/facilitators to maximising PRO impact; and iv) to examine real-world evidence of PRO trial data impact based on Research Excellence Framework (REF) impact case studies.

**Methods:**

Two independent investigators searched MEDLINE, EMBASE, CINAHL+, HMIC databases from inception until December 2018. Articles were eligible if they discussed research impact in the context of PRO clinical trial data. In addition, the REF 2014 database was systematically searched. REF impact case studies were included if they incorporated PRO data in a clinical trial.

**Results:**

Thirty-nine publications of eleven thousand four hundred eighty screened met the inclusion criteria. Nine types of PRO trial impact were identified; the most frequent of which centred around PRO data informing clinical decision-making. The included publications identified several barriers and facilitators around PRO trial design, conduct, analysis and report that can hinder or promote the impact of PRO trial data. Sixty-nine out of two hundred nine screened REF 2014 case studies were included. 12 (17%) REF case studies led to demonstrable impact including changes to international guidelines; national guidelines; influencing cost-effectiveness analysis; and influencing drug approvals.

**Conclusions:**

PRO trial data may potentially lead to a range of benefits for patients and society, which can be measured through appropriate impact metrics. However, in practice there is relatively limited evidence demonstrating directly attributable and indirect real world PRO-related research impact. In part, this is due to the wider challenges of measuring the impact of research and PRO-specific issues around design, conduct, analysis and reporting. Adherence to guidelines and multi-stakeholder collaboration is essential to maximise the use of PRO trial data, facilitate impact and minimise research waste.

**Trial registration:**

**Systematic Review registration** PROSPERO CRD42017067799.

## Introduction

Patient-reported outcomes (PROs) are increasingly used in clinical trials to assess the impact of a medical treatment or intervention. PRO assess a range of outcomes including symptoms, functional health, well-being and psychological issues from the patients’ perspective, without interpretation by a clinician [1–3]. Between 2007 and 2013, 26,337 (27%) of the clinical trials registered on ClinicalTrials.gov included PROs [4]. However, there is growing evidence that there is substantial research waste in relation to PROs [5, 6]. A recent systematic evaluation of oncology clinical trials determined that PRO protocol items were frequently omitted, non-reporting of PRO trials results was common and PRO publications were considerable delayed and presented suboptimal standards of reporting [5]. Thus, important PRO evidence may not be available to benefit patients and society.

Assessing the impact of research is a complex activity; however, it is important that the impact of PRO data is understood as this may inform funding allocations and demonstrate accountability to government, stakeholders and society [7]. Impact is defined as “*any identifiable benefit to, or positive influence on, the economy, society, public policy or services, culture, the environment or quality of life …*” *(p.26)* [8]. A number of reviews describe potential pathways (i.e. “*a way of achieving a specified result; a course of action’* [9]) to general research impact [8, 10–33]. However, few studies have investigated the optimal pathway or methods for augmenting or evaluating specific impacts of PRO trial data or the extent to which PRO impact is being realised. It is also not clear which is the most appropriate way to measure PRO impact, or the barriers and facilitators to realising that impact.

One way of assessing real-world impact is via the United Kingdom (UK) Higher Education Funding Council for England Research Excellence Framework (REF) impact case studies. During the REF 2014 exercise, UK higher education institutions submitted impact case studies: narratives that described the impact of research conducted during a specific time-period, including a number of case studies describing clinical trials involving PROs. REF case studies present meaningful, far-reaching and properly articulated impact that is demonstrated through convincing evidence. The impact presented focuses on the benefits of the research rather than on the pathways of research impact, allowing the assessment of real-world impact on society [34]. Examination of these case studies can enhance understanding of the best methods for maximising and measuring PRO research impact, not only in the UK, but also internationally since a number of the studies described in REF are international studies [10, 35].

Therefore, the study had four objectives. First, to conduct a systematic review of the literature to: i) determine the range of potential impact that may arise from clinical trial PRO clinical trial data, ii) identify potential PRO impact metrics iii) identify barriers/facilitators to maximising PRO impact and; iv) to examine REF 2014 impact case studies to explore real-world evidence of PRO trial data impact.

## Methods

This systematic review was registered on the PROSPERO database (CRD42017067799) and results are reported in accordance with PRISMA guidelines [36].

### Search strategy

#### Systematic review

Two reviewers (SCR and OLA) systematically and independently searched MEDLINE (Ovid), EMBASE, HMIC and CINAHL+ databases (inception to December 2018) for articles discussing the impact of PRO data collected from clinical trials from inception to December 2018 (see Additional file 1 for the full search strategy). The authors (SCR/MC/DK) designed the search strategy with input from a University of Birmingham Information Specialist. In addition, the keywords ‘patient reported outcome measure*’, ‘PROs’, ‘PRO’, ‘PROM’, ‘PROMS’, ‘HRQOL’, ‘HRQL’, ‘quality of life’, ‘impact’ and ‘clinical trial*’ were searched on Google Scholar, where the initial 100 results were screened. Only the first 100 results (10 pages) were revised, as article relevance diminishes with each page of results [37]. Lastly, additional publications (*n* = 3) were sought through communication with methodological PRO experts facilitated by MC/DK. Hand-searching of reference lists and citation searches of the included publications was also conducted to identify additional relevant articles.

#### REF 2014 impact case studies

The keywords “trial*” and “quality of life” or “patient reported outcome*” were introduced in the REF 2014 database. The search strategy was restricted to: i) Unit of assessment: main panel A (see Table 1 for further detail), ii) Summary impact type: ‘health’ and iii) Research subject area: medical and health sciences.

**Table 1:** REF 2014 – Main panel A

Units of assessment		
Main panel A	1	Clinical medicine
	2	Public Health, Health Services and Primary Care
	3	Allied Health Professions, Dentistry, Nursing and Pharmacy
	4	Psychology, Psychiatry and Neuroscience
	5	Biological Sciences
	6	Agriculture, Veterinary and Food Science

#### Eligibility criteria

Systematic review publications were deemed eligible if they discussed research impact in the context of PRO clinical trial data. In particular, we sought information on the types of impact (and pathways to impact) thought to be associated with PRO findings, proposed methods for measuring such impact and perceived barriers/facilitators to generating PRO-specific research impact. Publications were excluded if: i) solely focused on PROs used in routine clinical practice as the focus of this review was on the proposed PRO impact from trials; ii) trial publications reporting PRO results as the focus was research impact rather than primary results; or iii) conference abstracts. REF 2014 impact case studies were eligible if they included a trial in which PRO data were collected. There were no language restrictions.

### Data screening

#### Systematic review

The screening process was conducted independently by two reviewers (SCR and OLA). Citations were downloaded into Endnote® software (version X7.3.1) and duplicates deleted. Records were screened by title and abstract. Potentially relevant articles were identified for further full-text screening (SCR and OLA). Discrepancies were resolved through discussion with a third reviewer (MC/DK/AS) if required.

#### REF 2014 impact case studies

The screening process was also conducted independently by SCR and OLA. The case studies were downloaded into a Microsoft Excel spreadsheet. Records were screened by title and summary of the impact. Relevant case studies were selected for further full-text screening (SCR and OLA). Discrepancies were resolved through discussion, with a third reviewer (MC/DK/AS) as necessary.

### Data extraction/coding

#### Systematic review

Data extraction was done after the final selection of the included articles. SCR and OLA independently identified text excerpts that provided information on PRO-specific impact types, pathways, metrics, barriers or facilitators from the systematic review. Both reviewers independently imported text excerpts into a qualitative data analysis software package (QRS NVivo 11). They generated categories independently using descriptive coding under the directed content analysis framework [38]. The ‘pathways to research impact’ framework [10] was deductively applied to the data in order to identify types of impact and impact metrics. Data which did not fit within the existing framework were added to a ‘miscellaneous’ category. ‘Influence on policy-making’ was the only impact category discussed by the articles included in the systematic review. Subsequently, the data coded into this impact category was organised into subgroups. Through deductive coding, the following types of impact were identified: ‘inform clinical practice’, ‘inform clinical guidelines’, ‘inform clinical decision-making’, ‘inform health policy’ and ‘inform shared decision-making’.

Inductive coding was undertaken to describe and interpret more detailed codes within the ‘influence on policy-making’ and miscellaneous categories. The following types of impact were identified through inductive coding: ‘support drug approval’, ‘support pricing decisions’, ‘support reimbursement decisions’ and ‘inform consent for treatment’. In addition, inductive coding was used to identify further impact metrics, and barriers and facilitators to PRO trial impact. Development of overarching themes occurred after the coding process and collation of codes. The following details were also extracted from all the included publications: author, publication year, journal, methodology, study focus and type of PRO data impact.

#### REF 2014 impact case studies

Deductive and inductive was also undertaken to identify types of impact, impact metrics and barriers and facilitators among the REF case studies. In addition, the following details were extracted from the REF 2014 case studies: name, submitting institution and clinical area; trial name and year of publication, trial design, leading study centre, trial phase, trial primary and secondary outcomes, PRO instrument, significance of primary and secondary trial outcomes and type of impact. Furthermore, type of impact was further classified as either: i) direct PRO impact, where there was evidence of a direct link between PRO trial findings and subsequent impact. ii) Indirect PRO impact, where a trial including PROs subsequently led to impact, but it was not possible to directly attribute this impact to the PRO findings over and above the other trial outcomes; or iii) no evidence of PRO impact, where a trial including PROs failed to lead to impact. SCR and OLA independently piloted the coding frames, following discussion with MC/DK/AS/CM to resolve discrepancies. Finally, systematic review and impact case studies coding frames were validated by the co-authors MC/DK/AS/CM, who possess expertise in PRO clinical trial data, research impact and qualitative data analysis.

## Results

### Systematic review

#### Included studies

The search strategy retrieved 11,377 citations from MEDLINE (Ovid), EMBASE, HMIC, and CINAHL+; 100 citations were returned using Google Scholar and 6 through expert communication (PRISMA flow diagram, Additional file 2). Eight thousand eight hundred seventy-seven citations were excluded following review of title and abstract. In total, 32 full-text publications were assessed. Sixteen articles were excluded at this stage, as they assessed PRO data in routine care as an intervention. An additional 23 articles were included following hand-searching of reference lists and citation searches. In total, 39 eligible publications were included in the synthesis.

#### Study characteristics

The characteristics of the 39 included publications are summarised in Appendix 1. Fifteen (38%) publications were classified as systematic reviews, 11 as literature reviews (28%), eight (20%) as commentaries, three as qualitative studies and two as guidance papers. Non-English publications were identified through the different bibliographic methods used.

#### PRO impact types and pathways to impact

**Fig. 1:**
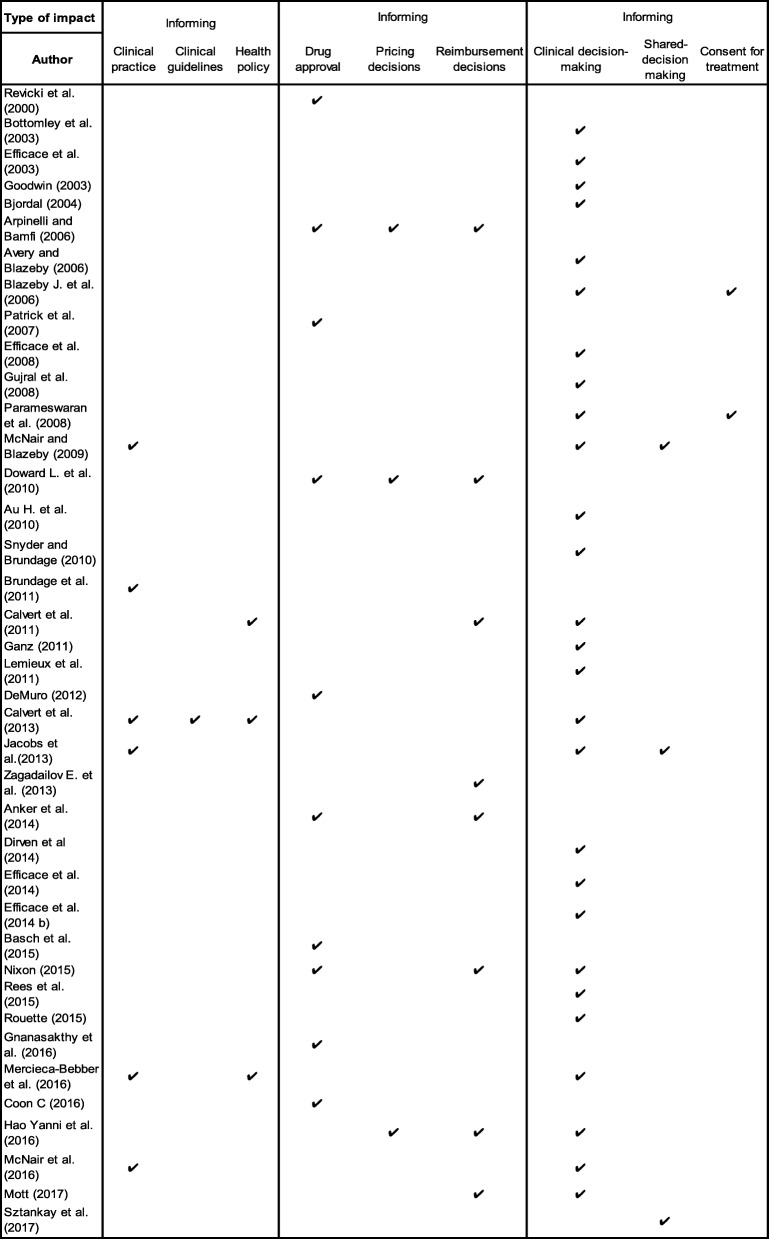
Proposed PRO impact types

The included publications identified nine types of impact that authors proposed could be associated with PRO trial findings. These included; ‘informing clinical practice’, ‘informing clinical guidelines’, ‘informing health policy’, ‘supporting drug approval’, ‘supporting pricing decisions’ and ‘supporting reimbursement decisions’, ‘informing clinical decision-making’ and ‘informing shared decision-making’ and ‘informing consent for treatment’ (Fig. 1).

The majority of publications (69%) focused on the potential impact of PRO trial results on clinical decision-making [7, 39–67]. Clinical decision-making refers to the clinical evidence-based decisions made regarding individual patient care by the clinicians whilst considering clinician’s knowledge, skills and attitudes, resources available and the patient’s own concerns, values and preferences [68]. 15% of the publications considered the benefits of using PRO trial results to inform clinical practice [53, 56, 62–64]. Clinical practice refers to the healthcare services provided to patients at an organisational level, which are normally adopted following clinical practice guidelines [69].

One paper focused on the impact of PRO trial results on clinical guideline development [46] and one paper focused on PRO data use in the development of healthcare policy [7]. Several publications recognised the impact of PRO trial findings on drug approval (29%) [49, 66, 70–76], pricing (7%) [61, 72, 77] and reimbursement decisions (20%) [7, 65, 66, 72, 75, 77, 78]. Eight publications (20%) discussed the influence of PRO data on pharmaceutical labelling claims by the Food and Drug Administration (FDA) and European Medicines Agency (EMA) [49, 66, 70–76]. An example given by one author was ruxolitinib (Jakafi™): an oral inhibitor to treat intermediate or high risk patients with myelofibrosis. This was the first FDA approved oncology drug that used PROs as an endpoint and followed FDA guidance to support a PRO-based labelling claim [78]. The oncology drug was approved based on reduction in spleen volume and improvement in symptom severity (e.g. weight loss, night sweats, itching, abdominal pain/discomfort, bone pain, cough, inactivity, early satiety and fever), as measured by the Myelofibrosis Symptom Assessment Form (MFSAF) v2.0 Total Symptom Score.

Lastly, one paper discussed the impact of PRO data on shared decision-making [79] and two papers (2%) on informing consent for treatment [44, 54]. The types of potential PRO impact proposed in each publication are summarised in Fig. 1 and described in Appendix 2. Shared decision-making is also important and four publications outlined the potential benefits of including PRO findings alongside other outcomes such as survival. This allowed patients and their clinicians to make an informed joint decision about treatment preferences and symptom management based on mutual understanding of treatment objectives and expectations [1–4].

#### PRO impact metrics

Based on the review of the 39 included publications, two impact metrics were identified. Number of pharmaceutical labelling claims approved, mentioned by eight authors [70–76, 80] and number of promotional labelling claims, discussed by one author [70].

#### Barriers to impact

Authors identified different perceived methodological barriers to PRO trial impact, which fell within the following categories: ‘trial design’, ‘conduct and analysis’, ‘reporting’ and the ‘use of PRO data in practice’.

#### Trial design

Suboptimal PRO-specific trial design was cited by a large number (*n* = 22, 56%) of publications as a major barrier to realising PRO trial impact [40–43, 45–47, 49, 51, 52, 55, 62–64, 70, 72, 73, 76, 78, 81–83]. Particular areas of concern were selection of inappropriate or invalid PRO measures (*n* = 10, 52%). Lack of development of PRO cross-cultural items (*n* = 2, 9%). Broader methodological issues leading to potential bias and which may hinder PRO data use include allocation concealment, randomisation, blinding of participants and personnel and blinding of outcomes assessment (*n* = 7, 18%).

#### Conduct and analysis

Authors also highlighted that the way the trial was conducted and type of analysis carried out could act as an impact barrier. Over a third of publications (*n* = 12, 31%) identified PRO-specific barriers associated with trial conduct and analysis [40, 41, 56, 57, 62, 63, 70, 72, 73, 75, 78, 83]. The most frequent barriers mentioned by authors were low PRO compliance rates (*n* = 10, 83%), lack of personnel training on administration of PRO instruments (*n* = 3, 25%), incomplete follow-up of HRQL assessment (*n* = 2, 16%), selection of inappropriate statistical methods to handle missing data (*n* = 2, 16%).

#### Reporting

Incomplete or suboptimal reporting of PRO trial data was cited by 23 (65%) publications as a barrier to research impact if PRO trial findings and generalisability is not clearly presented [7, 38–43, 45–53, 56, 57, 63, 64, 72, 82, 83]. The most common barriers to impact were failure to report: the rationale for the chosen PRO instrument (*n* = 10, 43%), mode of administration (*n* = 10, 43%).

#### Use of PRO data in practice

Adoption of PRO trial findings into clinical practice was identified as somewhat problematic by 17 (43%) of the publications [39–41, 43, 45, 46, 52, 54–56, 61, 63, 64, 75, 78, 81, 82]. Key issues included lack of training/practice for clinicians on interpreting PRO data (*n* = 11, 64%) and lack of familiarity with PRO measures (*n* = 7, 41%).

#### Facilitators to impact

The review of the included articles identified a number of suggested facilitators purported to enhance the probability of realising PRO specific impact. Two (5%) authors suggested strict adherence to the Standard Protocol Items: Recommendations for Interventional Trials (SPIRIT) initiative to improve the completeness of trial protocols and reduce risk of bias [51, 62]. Eight (18%) authors proposed adherence to the Consolidated Standards of Reporting Trials (CONSORT) PRO Extension statement [7, 51–53, 56, 62, 64, 83] and two (5%) authors to the CONSORT statement [7, 51], in order to enhance transparency and complete reporting of PRO clinical trials. A detailed summary of the barriers and facilitators highlighted by the included publication are presented in Table 2, which includes additional resources identified by communication with methodological PRO experts (MC/DK).

**Table 2:** Barriers and facilitators to maximising PRO trial data

Barriers to impact	Impact Facilitators
PRO trial design	
Authors not using/citing guidelines to design PRO trials [69, 75, 76]	●SPIRIT ●SPIRIT-PRO Ext^a^
Selection of inappropriate PRO time frames of assessment [38, 42, 44, 58]	●SPIRIT ●SPIRIT-PRO Ext^a^
Failure to define PRO/HRQL endpoints [47]	●SPIRIT-PRO Ext^a^
Selection of inappropriate or invalid PRO measures [42, 44, 50, 52, 54, 57, 60, 66, 67, 69]	●SPIRIT-PRO Ext^a^ ●ISOQOL Minimum Standards for PRO Measures in patient-centered outcomes and comparative effectiveness research^a^
Inappropriate PRO sample size and population [38, 48, 54, 56, 59]	●SPIRIT ●SPIRIT-PRO Ext^a^
Issues of bias due to allocation concealment (selection bias), random sequence generation (selection bias), blinding of participants and personnel (performance bias) and blinding of outcomes assessment (detection bias) [57, 68, 69, 75, 76, 79, 82]	●SPIRIT
Lack of evidence of PRO translation or cross-cultural validation [53, 57]	●SPIRIT-PRO Ext^a^
PRO trial conduct and analysis	
Low PRO compliance rates [38, 39, 42, 44, 50, 60, 61, 77, 79, 82]	●SPIRIT-PRO Ext^a^ ●SISAQOL^a^
Lack of personnel training on administration of PRO instruments [44, 57, 61]	●SPIRIT-PRO Ext^a^ ●SISAQOL^a^
Lack of communication between researchers and administrators regarding PRO questionnaires involved in the trial [44]	●SPIRIT-PRO Ext^a^ ●SISAQOL^a^
Lack of standardisation of the PRO questionnaire administration process [44, 61]	●SPIRIT-PRO Ext^a^ ●SISAQOL^a^
Lack of patient adherence to the PRO component of the study due to questionnaire length or irrelevant content [44, 52, 61]	●SPIRIT-PRO Ext^a^ ●SISAQOL^a^
PRO trial reporting	
Authors not using/citing guidelines to report PRO trials (e.g. CONSORT PRO Extension) [54, 69, 75, 76]	●CONSORT ●CONSORT-PRO Ext
Failure to report the a priori PRO hypothesis [39, 50, 54, 58, 59, 62, 63, 69]	●CONSORT ●CONSORT-PRO Ext ●SPIRIT-PRO Ext
Failure to report baseline PRO compliance [39, 50, 59, 62, 69]	●CONSORT
Failure to report rationale for the chosen PRO instrument [7, 39, 44, 50, 54, 58, 62, 69, 72, 76]	●CONSORT-PRO Ext ●SPIRIT-PRO Ext
Failure to report mode of administration of the PRO instrument [44, 47, 48, 50, 54, 58, 62, 63, 75, 76]	●CONSORT-PRO Ext ●SPIRIT-PRO Ext
Failure to report timing of PRO assessment [37, 58, 59]	●CONSORT ●SPIRIT-PRO Ext
Failure to report methods of PRO data collection [62, 63]	●CONSORT ●CONSORT-PRO Ext
Failure to report clinical significance of PRO findings [39, 40, 47, 56, 59, 62, 67, 75]	CONSORT-PRO Ext
Reporting levels of missing PRO data [7, 39, 52, 58, 59, 62]	●CONSORT ●CONSORT-PRO Ext
Failure to report statistical methods dealing with missing PRO data [39, 54, 56, 58, 62, 63, 69, 75]	●CONSORT ●SPIRIT-PRO Ext
Failure to report generalisability of PRO trial results in the context of clinical outcomes [54, 56, 69, 76, 82]	CONSORT-PRO Ext
Selective reporting of PRO results [7, 75, 76]	●CONSORT ●SPIRIT-PRO Ext
Discrepancies between PRO protocol and PRO trial report [44]	●CONSORT ●SPIRIT-PRO Ext
Failure to report PRO data in the main trial publication [47, 48, 54, 59, 63, 72]	●Publication of HRQL and other clinical outcomes in the main trial report [48, 67, 69, 72]
Late publication of PRO trial results and in a different journal to the main publication [42, 48, 56, 67, 72, 77]	●Publication of secondary and timely PRO publication [63, 69]
Journal word restrictions [54, 69]	●Journals should allow space to report HRQL data alongside other clinical outcomes [50]
Barriers to uptake of PRO trial results in practice	
Lack of familiarity with PRO measures [42, 44, 45, 50, 60, 67, 71]	●PROlearn^a^ ●SPIRIT-PRO Ext ●Provide training to clinicians to gain confidence regarding the validity and reliability of HRQL instruments [67]
Lack of training/guidance for clinicians on interpreting PRO data [40, 42, 44, 45, 48, 50, 53, 58, 66, 67, 69]	●PROlearn^a^ ●Training for clinicians to understand clinical interpretation of HRQL data [48, 50] ●Clinician’s checklist for reading and using an article about patient-reported outcomes^a^
Clinicians concerns about the PRO results being biased by missing data [77]	●PROlearn^a^ ●Provide training to clinicians to gain confidence regarding the validity and reliability of HRQL instruments [67] ●Clinician’s checklist for reading and using an article about patient-reported outcomes^a^
Lack of evidence of generalisability of PRO/HRQL results [42, 53, 67, 71]	●CONSORT ●Clinician’s checklist for reading and using an article about patient-reported outcomes^a^
Concerns that the PRO results were chance findings arising from multiple testing [77]	●PROlearn^a^ ●Provide training to clinicians to gain confidence regarding the validity and reliability of HRQL instruments [67] ●Clinician’s checklist for reading and using an article about patient-reported outcomes^a^
Researchers failure to present PRO data in a way that is accessible to patients and clinicians [54, 69]	Use of graphical methods to present PRO results [42, 44, 48, 50] ●Stakeholder-driven, evidence-based standards for presenting PROs in clinical practice^a^
Lack of time to discuss PRO outcomes with patients [67]	●PROlearn^a^ ●Provide consistent and improved HRQL data reports and a summary of the clinical implications of the HRQL results [67] ●Provide training to clinicians to gain confidence regarding the validity and reliability of HRQL instruments [67]
Overburden of staff, clinicians, participants and resources [42, 44, 56, 61]	●SPIRIT-PRO Ext^a^

ISOQOL Minimum Standards for PRO Measures in patient-centred outcomes and comparative effectiveness research [83]. CONSORT (Consolidated Standards of Reporting Trials) [84]. CONSORT-PRO Extension [58]. SPIRIT (Standard Protocol Items: Recommendations for Interventional Trial) [85]. SPIRIT-PRO Extension [3]. SISAQOL (The Setting International Standards in Analyzing Patient-Reported Outcomes and Quality of Life Endpoints Data) [86]. Stakeholder-driven, evidence-based standards for presenting PROs in clinical practice [87]. Clinician’s checklist for reading and using an article about patient-reported outcomes [88]. PRO Learn [89]. *Ext* Extension

^a^Additional resources identified through expert communication

#### REF 2014 impact case studies

Examples of clinical trial PRO impact were explored using REF2014 case studies. These identified a range of impact metrics.

#### Included studies

The search strategy yielded 209 REF 2014 impact case studies (PRISMA Flow Diagram, Additional file 3). Case studies were excluded if they did not include a clinical trial or the clinical trials did not incorporate a PRO element, meaning 69 relevant case studies were subsequently included in the analysis.

#### PRO clinical trials characteristics

The characteristics of the PRO clinical trials included across the eligible REF 2014 case studies are detailed in Table 3.

**Table 3:** PRO clinical trials characteristics

Trial characteristics	Number of trials, (%)
Trial phase	
I	0
I/II	1 (1.4)
II	1 (1.4)
III	24 (34)
Other	3 (5.7)
Not specified	40 (57)
Leading study centre	
UK	62 (89)
International	7 (11)
Trial design	
International multicentre study	21(30)
PRO outcome	
Primary outcome	17 (24)
Secondary outcome	35 (50)
Both	11 (15)
PRO measures used	
SF-36	17 (24)
EQ-5D	12 (17)
HADS	9 (13)
VAS	9 (13)
EORTC QLQ-C30	3 (4)
Other	70^a^

^a^Number of different PRO measures identified – eCase studies characteristics

**Fig. 2:**
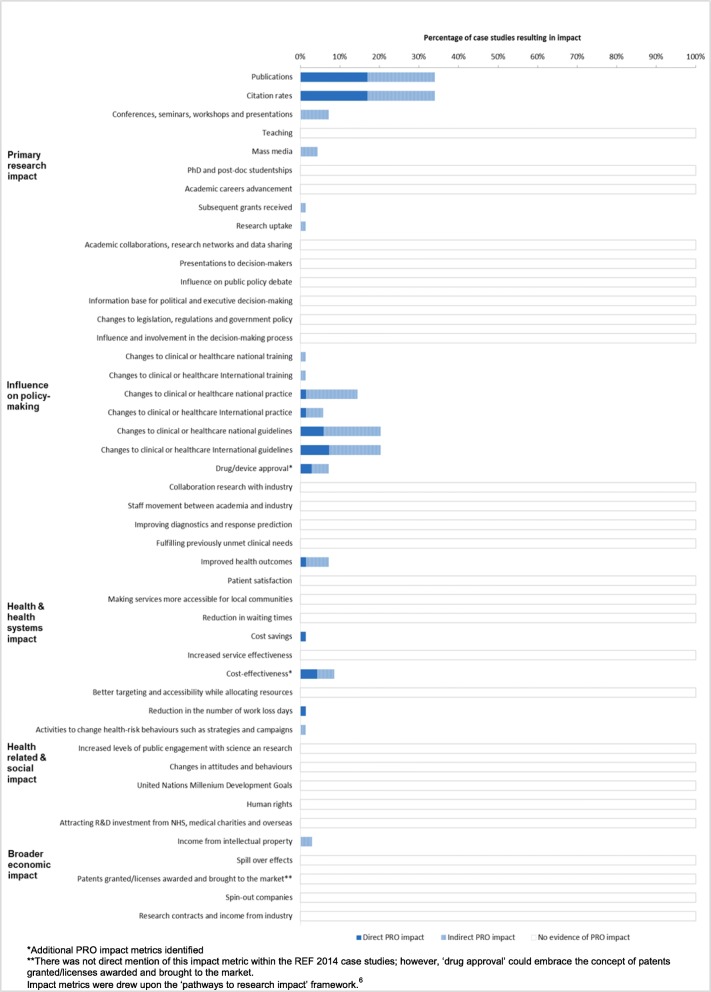
PRO trial impact metrics. *Additional PRO impact metrics identified. **There was not direct mention of this impact metric within the REF 2014 case studies; however, ‘drug approval’ could embrace the concept of patents granted/licenses awarded and brought to the market. Impact metrics were drew upon the ‘pathways to research impact’ framework [6]

Full details of the included case studies are available in Additional file 4. The assessment of the PRO trial metrics was considered using the ‘pathways to research impact’ framework [10]. Following this, two new additional impact metrics were identified, cost-effectiveness and drug/device approval. The summary of the PRO impact metrics is depicted in Fig. 2.

#### Real-world evidence of PRO impact

Assessment of the 69 eligible case studies determined that (*n* = 12, 17%) appeared to lead to direct demonstrable PRO impact, (*n* = 12, 17%) showed evidence of indirect PRO impact and (*n* = 45, 66%) provided no evidence of PRO impact (Fig. 2). Trials that included PROs as primary outcome (50%) reported a larger number of trials leading to direct impact than those trials that had PROs as secondary outcome (83%).

#### Direct PRO impact

The most common types of direct PRO impact presented across the case studies included: number of publications (*n* = 12, 17%), citation rates (*n* = 12, 17%), changes to international guidelines (*n* = 5, 7%), contribution to national guidelines (*n* = 4, 6%), contribution to evidence of cost-effectiveness (*n* = 3, 4%) and informing drug approval (*n* = 2, 3%). In addition, several case studies demonstrated more than one type of impact.

#### Indirect PRO impact

The most common types of indirect PRO impact included: number of publications (*n* = 12, 17%), citation rates (*n* = 12, 17%), changes to national guidelines (*n* = 10, 14%), contribution to international guidelines (*n* = 9, 13%) and national practice (*n* = 9, 13%) and contribution to evidence presented in conferences, seminars and workshops (*n* = 5, 7%).

#### Absence of evidence around PRO impact

The assessment of the included case studies demonstrated that the impact of PRO trial data is not usually captured in the long-term, specifically under the impact categories health and health system impacts, health related and social impact and economic impact.

## Discussion

This manuscript is the first to present a systematic review aimed at identifying the potential types of PRO trial impact alongside real-world evidence of impact. It will allow researchers, clinicians, funders and policy-makers to consider pathways to research impact before conducting PRO trial research and to identify metrics to assess impact prospectively. In the same way, it will allow PRO stakeholders to consider facilitators at the design, conduct, analysis and reporting stages whilst avoiding recurrent barriers to generating PRO-specific impact and minimising research waste. High quality clinical trials involving PROs may lead to benefits for patients and society.

Nine types of potential PRO trial impact were identified (Fig. 1): informing clinical practice, informing clinical guidelines, informing health policy, supporting drug approval, supporting pricing and supporting reimbursement decisions, informing clinical and shared decision-making and informing consent for treatment. Only four impact metrics were proposed to measure the impact of PRO data, number of pharmaceutical claims and promotional labelling claims and inform drug/device approval and cost-effectiveness. Further research to formalise PRO-specific impact metrics is required.

Authors suggested that potential barriers to the use of PRO trial findings to inform healthcare decision-making and patient care included poor quality trial design, conduct, analysis, reporting and uptake in practice [55]. Several of the barriers comprised within ‘uptake of PRO trial results in practice’ (Table 2) are not unique to PRO clinical trials. These challenges are also encountered in the implementation of PRO data collected in routine clinical practice to inform patient care or for audit/benchmarking purposes. For instance, ‘high levels of missing data’ ‘overburden of staff, clinicians, participants and resources’, ‘lack of training/guidance for clinicians on interpreting PRO data’ are challenges commonly faced in routine practice [84, 85]. Furthermore, it is important to note that many of these challenges are not unique to PRO data/trials. Greater efforts are required to improve outcome selection, collection and reporting in both in trials and routine care [86, 87].

Suboptimal reporting of PRO trial data was the most discussed barrier (65%), which might hinder the maximisation of PRO trial findings. Therefore, addressing poor and incomplete reporting is essential, as it is unethical to waste research funding, resources and patients’ efforts and time invested during the collection PRO trial data [5, 6]. In recent years, a number of methodological guidelines have been developed to address the different barriers highlighted by this systematic review. These include: the SPIRIT-PRO Extension to improve the completeness of trial protocols [3]; The ongoing work of the SISAQOL Consortium to standardise the analysis and interpretation of PRO and quality of life from oncology clinical trials [88]; CONSORT-PRO Extension to facilitate optimal reporting guidance of trials that include PROs as primary or secondary outcome [46] and; the work carried out by Snyder et al. (2017) to present PRO trial findings [89]. The adoption of these guidelines has the potential to improve the design, conduct, analysis and report of PRO trials thus ensuring that high-quality data that may benefit patients and society are obtained from trials. The uptake of these guidelines is currently being promoted through PROTEUS (Patient-Reported Outcomes Tools: Engaging Users & Stakeholders) Consortium, which is funded by the US Patient Centred Outcomes Research Institute (PCORI) [90].

The literature suggests that adherence to guidelines should be endorsed/mandated by journals/editors in order to ensure high quality PRO data through the trial design, implementation, analysis and reporting stages. In addition, the FDA [2] and EMA [91] provide guidance to sponsors on reporting of PRO instrument development, measurement properties, implementation, analysis, and interpretation used to support drug approval and pharmaceutical labelling claims in the United States and Europe, respectively. Additional facilitators identified to maximise the realisation of impact in practice were reporting of PRO results adequately within the main trial publication, whilst considering journals word restrictions [43, 52, 53, 56, 64] and clinicians receiving training/guidance on interpretation of PRO data [41, 43, 52, 63]. Thus, it is essential that funders, ethics committees, journal editors and trial researchers proactively work together to ensure that PRO studies follow optimal design, conduct and analysis and reporting.

Although the systematic review publications may not have included information on PRO trial data impact, we explored whether evidence of impact could be identified from REF 2014 impact case studies as by their nature, they present an opportunity for researchers to highlight the impacts of their research. Sixty-nine REF 2014 case studies included a trial where PRO data were collected. Of these, 24 (34%) presented evidence of PRO trial impact that was classified as direct or indirect impact. Direct attribution of impact to PRO trial data was possible in 12 trials, most commonly informing national and international clinical guidelines. A number of potential impact categories are currently unrealised or under reported. This could be attributed to the fact that some of the PRO trials associated to the case studies have been published in the last years, which limits demonstration of PRO trial impact in the mid and long-term [10]. Furthermore, it was often difficult to unpick the exact contribution of PRO data to this impact as they were commonly combined with ‘clinical’ outcome data.

**Fig. 3:**
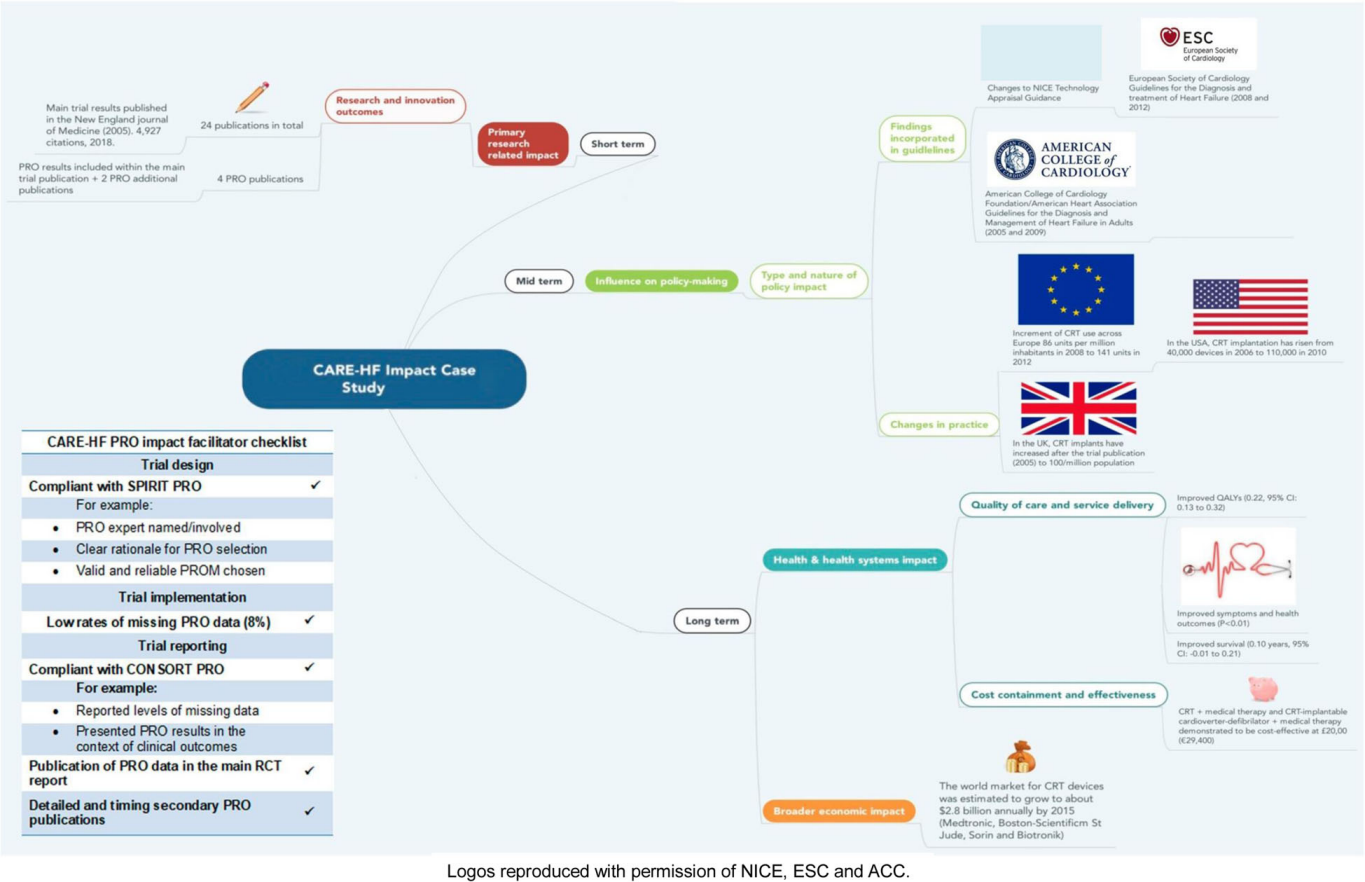
CARE-HF Trial Pathways to PRO Trial Impact. Logos reproduced with permission of ESC and ACC

The REF 2014 case study ‘Heart failure: Improving the quality of life and survival of heart failure patients through Cardiac Resynchronisation Therapy’, submitted independently by the University of Birmingham [92] and Hull [93] is described below (Table 4) to illustrate the different facilitators that might help translating PRO findings into clinical practice. This example was chosen, as it is one of the case studies that have led to most varied impact and will provide researchers a useful guide about how to maximise PRO trial data and reduce research waste (Fig. 3).

**Table 4:** Practical guide for researchers

The Cardiac Resynchronisation — Heart Failure (CARE-HF) trial demonstrated that the cardiac resynchronisation therapy reduced the risk of complications and death among patients with left ventricular systolic dysfunction and cardiac dyssynchrony who had moderate or severe heart failure [85, 86]. In addition, as measured with the EQ-5D and Minnesota Living with Heart Failure Questionnaire (MLWHF), the therapy was associated with quality of life and symptoms improvement [86]. ●The main trial publication was characterised for complying with the different facilitators identified by the systematic review and for adhering to the SPIRIT PRO Extension and CONSORT PRO Extension guidelines despite these guidelines being published subsequently. ●Low rates of PRO missing data (8%) and statistical methods for dealing with missing data were reported. ●The PRO data was included in the main RCT report and alongside other clinical data [87]. In addition, there were detailed and timely secondary PRO publications [86, 88, 89]. Attributing impact directly to PRO data is difficult given the survival benefit; however, this well designed, conducted, analysed and reported trial led to impact that could be measured through the following impact metrics: ●In the short term, PRO results were included in the main trial publication, [87] which led to 4927 citations by January 2018. At least 4 additional PRO trial publications are available. ●In the mid-term, PRO trial findings were incorporated in clinical guidelines and health policy at national and international level: NICE in the UK, [90] the European Society of Cardiology, [91] the European Society of Cardiology in Canada, [92] Brazil, [93] and USA [94]. Therefore, the use of CRT influenced the healthcare practice at national and international level by providing the CRT to patients with heart failure and dyssynchrony. ●In the long-term, an additional study assessing the effects of the CARE-HF trial on quality of life demonstrated that the device improved quality of life and symptoms and improved survival among the users [88]. In addition, PRO results informed the cost-effectiveness analysis of the intervention and the production of the device, [89, 95] which led to increased income from industry: *‘the world market for CRT devices is projected to grow to $2.8 billion annually by 2015’.* [81] The cost-effectiveness analysis demonstrated that CRT is cost-effective when compared with medical therapy alone (MT). In the same way, CRT plus cardioverter-defibrillator is more cost-effective when compared to CRT + MT.[95]

However, reported barriers and facilitators in the literature focused predominantly on the PRO clinical trial design, conduct and analysis stages. There was a dearth of information on how to address barriers to generating PRO trial impact in practice. Additionally, identified impact was mainly focused on primary research (e.g. publications, citations and conference). There was little attention on policy-making, health & health systems, health related & societal and economic impact, which is generally realised in the mid and long term. Thus, further research in this area is required to identify facilitators to maximise PRO trial impact in the longer term. This will be achieved through interviews with international stakeholders in order to explore in-depth perceived barriers and facilitators to effective dissemination and impact on healthcare decisions and patient care. Work will be conducted to refine the ‘pathways to research impact’ framework in the context of PRO trial impact. In addition, it is important to mention that it is well established that certain impact categories (e.g. primary research impact via publications) are easier to measure than others (e.g. societal impact), which can limit the number of available impact metrics to measure the impact of PRO trial data.

### Limitations

This systematic review summarised the different types of impact thought to be associated with PRO trial findings and proposes metrics to measure impact in practice. The main limitation was that due to poor indexing, over half of the included publications were identified through hand-searching of references lists and citation searches methods rather than databases searching. Therefore, some relevant publications might not be included in this article if they failed to mention a type of impact in the title/abstract. However, we made efforts to identify all the relevant publications. The search strategy adopted did not include the search term ‘self-rated health’, which could have led to the exclusion of relevant articles. Nonetheless, our search strategy was informed by the Oxford PROM Group Construct & Instrument Type Filter [94], which was modified according to the objectives of this systematic review. Although there were no language restrictions, we did not systematically search non-English databases. In addition, a formal quality appraisal was not undertaken to assess the quality of the studies included. We acknowledge that a significant amount of the evidence we found was based on expert opinion, which does not rank highly in the evidence hierarchy. Furthermore, a small number of the included studies were discussed by different authors, which may have influenced the frequency counts. However, this does not affect the conclusions of this systematic review.

The ‘pathways to research impact’ framework was used to measure the impact of PRO trial data. This framework was selected as it synthetises all the existing types of healthcare research impact and metrics (Fig. 2). However, not all the types of impact outlined by the framework are relevant to PRO trial data (e.g. human rights and United Nation Millennium Development Goals). The REF is an expert review process solely focused on the UK HEIs, which may limit the generalisability of the impact of the PRO data, although 30% of the trials were categorised as international trials. In some instances, it was not possible to confirm the impact described by the REF 2014 impact case studies, as there was no access to some sources provided. It is important to consider that the case studies had word count restrictions, which could have led to under reporting of impact. In addition, the majority of the articles included in the first section of the systematic review focused on oncology. Therefore, the findings presented in this study can only be generalised to oncology PRO clinical trials.

## Conclusion

This review provides a summary of the different types of potential PRO impact identified in the literature, supported by real-world examples. The impact of PRO clinical trials can be attributed to PRO results and measured through different impact metrics. It is essential that researchers and authors design, conduct and analyse and report high quality PRO trial results and; proactively tackle barriers to PRO impact in order to maximise the impact of PRO clinical trials in the short, mid and long term to fully realise benefits for society. Adherence to guidance and multi-stakeholder collaboration is essential to maximise the utilisation of PRO trial data, while minimising research waste and maximising future patient care.

### Supplementary information

**Additional file 1.** Search strategies

**Additional file 2.** Systematic Review PRISMA Flow Diagram

**Additional file 3.** REF 2014 Impact case studies PRISMA flow diagram

**Additional file 4.** REF impact case studies

## Data Availability

All data generated or analysed during this study are included in this published article [and its supplementary information files].

## Appendix 1

**Table 5:** Study characteristics of the literature review

Author	Journal	Publication type	Publication focus	Types of PRO impact discussed
Revicki et al. (2000) [38]	Quality of Life Research	Guidance paper	Recommendations on use of HRQL data to support labelling and promotional claims	Informing drug approval
Bottomley et al. (2003) [39]	American Society of Clinical Oncology	Systematic review	HRQL in Non-small-cell lung cancer	Influencing clinical decision-making
Efficace et al. (2003) [40]	Journal of Clinical Oncology	Guidance paper	A checklist for evaluating HRQL in prostate cancer trials	Informing clinical decision-making
Goodwin et al. (2003) [41]	Journal of the National Cancer Institute	Literature review	HRQL in breast cancer trials	Informing clinical decision-making
Bjordal (2004) [42]	Annals of Oncology	Literature review	Impact of HRQL assessments within trials on clinical practice	Informing clinical decision-making
Arpinelli and Bamfi (2006) [43]	Health and Quality of Life Outcomes	Commentary	PRO trial data in drug development	Informing drug approval Informing reimbursement decisions Informing pricing decisions
Avery and Blazeby (2006) [44]	World Journal Surgery	Systematic review	HRQL in breast, prostate, lung and colorectal cancer trials	Informing clinical decision-making
Blazeby et al. (2006) [45]	Journal of Clinical Oncology	Literature review	HRQL in surgical oncology trials	Informing clinical decision-making Influencing informed consent
Patrick D. et al. (2007) [46]	Value in Health	Literature review	Use of PRO data to support medical product labelling claims (FDA perspective)	Informing drug approval
Efficace et al. (2008) [47]	European Journal of Cancer	Systematic review	HRQL in leukaemia trials	Informing clinical decision-making
Gujral et al. (2008) [48]	Support Care Cancer	Systematic review	Quality of life after colorectal cancer surgery	Informing clinical decision-making
Parameswaran et al. (2008) [49]	Annals of Surgical Oncology	Systematic review	HRQL in surgery for esophageal cancer	Influencing clinical decision-making Influencing informed consent
McNair and Blazeby (2009) [50]	Expert Reviews Pharmaeconomics Outcomes Research	Literature review	HRQL in gastrointestinal cancer trials	Informing clinical practice Informing clinical decision-making Inform shared decision-making
Au H. et al. (2010) [51]	Expert Review of Pharmacoeconomics & Outcomes Research	Review	HRQL in oncology clinical trials	Informing clinical decision-making
Doward L. et al. (2010) [52]	Health and Quality of Life Outcomes	Commentary	Use of PRO trial data to inform pharmaceutical labelling claims and payers	Informing drug approval Informing pricing decisions Informing reimbursement decisions
Snyder and Brundage (2010) [53]	Expert Reviews Pharmaeconomics Outcomes Research	Commentary	PROs in healthcare policy, research and practice	Informing clinical decision-making
Brundage et al. (2011) [54]	Quality of Life Research	Systematic review	PROs in Phase III randomised clinical trials	Informing clinical practice
Calvert et al. (2011) [7]	The Lancet	Systematic review	Quality of life in clinical trials	Informing clinical decision-making Informing health policy Informing drug approval
Ganz (2011) [55]	Journal of the National Cancer Institute	Commentary	Quality of life measurement in breast cancer trials	Informing clinical decision-making
Lemieux et al. (2011) [56]	Journal of the National Cancer Institute	Systematic review	Quality of life in breast cancer trials	Influencing clinical decision-making
DeMuro et al. (2012) [57]	Value in Health	Literature review	Reasons why PRO label claims were rejected and provide feedback from the regulatory perspective regarding the use of PROs in clinical trials	Informing drug approval
Calvert et al. (2013) [58]	Health and Quality of Life Outcomes	Commentary	Implications of the CONSORT PRO extension on clinical trials and practice	Informing clinical practice Informing clinical guidelines Informing health policy Informing clinical decision-making
Jacobs et al. (2013) [59]	Quality of Life Research	Systematic review	HRQL in oesophageal cancer trials	Informing clinical practice Informing clinical decision-making Informing shared decision-making
Zagadailov E. et al. (2013) [60]	American Health & Drug Benefits	Literature review	Challenges and opportunities of incorporating oncology PRO trial data into reimbursement decisions	Informing reimbursement decisions
Anker et al. (2014) [61]	European Heart Journal	Literature review	Cardiovascular PRO clinical trials	Informing drug approval Informing reimbursement decisions
Dirven et al. (2014) [62]	European Journal of Cancer	Systematic review	PROs in brain tumour trials	Influencing clinical decision-making
Efficace et al. (2014) [63]	European Association of Urology	Systematic review	PROs in prostate cancer trials	Informing clinical decision-making
Efficace et al. (2014b) [63]	European Journal of Cancer	Systematic review	PROs in gynaecological cancer trials	Informing clinical decision-making
Basch E. et al. (2015) [64]	JAMA Oncology	Qualitative study	PRO trial data in cancer drugs development	Informing drug approval
Nixon et al. (2015) [65]	Farmeconomia. Health Economics and Therapeutic Pathway	Commentary	PRO data to support drug development decision-making	Informing drug approval Informing reimbursement decisions Informing clinical decision-making
Rees et al. (2015) [66]	Journal of Cancer Research and Clinical Oncology	Systematic review	PROs in colorectal cancer trials	Informing clinical decision-making
Rouette et al. (2015) [67]	Quality of Life Research	Literature review	Oncologists’ perspectives on HRQL in trials among countries and specialities	Informing clinical decision-making
Gnanasakthy et al. (2016) [68]	Journal of Clinical Oncology	Literature review	PRO labelling for products approved by the Office of Haematology and Oncology Products of the FDA	Informing drug approval
Mercieca-Bebber et al. (2016) [69]	European Journal of Cancer	Systematic review	PROs in head, neck and thyroid cancer trials	Informing health policy Informing clinical practice Informing clinical decision-making
Coon C (2016) [70]	Clinical Therapeutics	Commentary	PRO oncology clinical trials	Informing drug approval
Hao Yanni et al. (2016) [71]	Clinical Therapeutics	Commentary	PRO oncology clinical trials	Informing reimbursement decisions Informing pricing decisions Informing clinical decision-making
McNair et al. (2016) [72]	PLOS One	Systematic review	PRO and clinical gastro-intestinal cancer data in trials	Informing clinical decision-making Informing clinical practice
Mott (2017) [73]	Oncology and Therapy	Qualitative study	PROs and lung cancer	Informing reimbursement decisions Informing clinical decision-making
Sztankay et al. (2017) [74]	BMC Cancer	Qualitative study	HRQL in patients with advanced non-small cell lung cancer	Informing shared decision-making

## Appendix 2

### Potential types of PRO impact proposed

#### Clinical practice, clinical guidelines and health policy

A number of authors discussed the potential influence of PRO data on clinical practice, clinical guidelines and health policy. Several authors felt inclusion of PRO data on clinical guidelines may influence clinical practice, by fulfilling unmet clinical needs and leading to improved patient centre care by helping patients make more informed decisions on their care [44, 48, 51, 58–60, 76]. Three studies suggested that the inclusion of PRO data in clinical guidelines might ensure wider acceptance of guideline recommendations among patients, while enhancing implementation through health policy [5, 51, 60].

#### Drug and device approval

Authors reported that PRO data is increasingly used to provide evidence for drug and device approval, especially in oncology clinical trials [67, 68]. Eight publications discussed the influence of PRO data on pharmaceutical labelling claims by the Food and Drug Administration (FDA) and European Medicines Agency (EMA) [44, 62, 64–70]. One publication suggested that PRO data may inform drug and device approval through communication of the benefits and harms of the intervention has to clinicians, patients and other consumers [64].

#### Pricing and reimbursement decisions

Three publications discussed the influence of PRO data on pricing decisions [57, 66, 71] and seven on drug reimbursement decisions [5, 57, 61, 66, 69, 71, 74]. Authors suggested that inclusion of PRO data provided valuable information regarding the risk-benefit of new interventions. Several authors postulated that within the context of oncology treatment, PRO data have the potential to identify costly events reported by the patients in the trials. Hence, interventions with reduced toxicity and added PRO benefit may influence payer decision-makers [72]. Additionally, the inclusion of patient advocacy groups may influence the availability of interventions, enhancing the patient healthcare experience by incorporating the ‘patient’s voice’ throughout payer decision-making [57, 66].

One such example illustrated in the literature, is the decision by NICE (2015) to recommend the use of nintedanib plus docetaxel (Vargatef®) as a treatment option for locally advanced, metastatic or locally recurrent non-small-cell lung cancer of adenocarcinoma histology. The drug approval was primarily based on improved survival, minimal adverse drug effects and fewer detrimental effects on health-related quality of life (HRQL) compared to chemotherapy treatment. PRO data suggested that HRQL, as measured by the EuroQol-5 Dimension (EQ-5D), the European Organization for Research and Treatment core quality of life questionnaire (EORTC QLC-C30) and the lung cancer–specific Quality of Life Questionnaire (EORTC QLQ-LC13) was similar in both groups [57, 96]. However, the PRO measures also demonstrated better pain management in patients randomised to nintedanib plus docetaxel based on information from the pain items [57, 96]. Therefore, additional drug benefits (symptom improvement and tolerability) were demonstrated through incorporation of PRO measurements into the trial, which similarly informed the reimbursement decision. The additional HRQL benefits may have been missed without the supporting PRO data within this trial.

#### Clinical decision-making

While reducing the impact of intervention toxicity and the impact on HRQL is important for drug labelling claims, it is also an important consideration for choices in clinical decision-making. According to Goodwin et al. (2003), when there is medical treatment equivalence, HRQL data has the potential to inform clinical decision-making by prioritising quality of life outcomes and reduction in toxicity when making clinical decisions [56]. The authors conducted a systematic review of breast cancer clinical trials including PROs and determined that HRQL contributed to clinical decision-making within primary management (surgery, radiation and hormone therapies) and symptom control/supportive care setting of breast cancer.

In total, 26 publications presented evidence that PRO data may help in the selection of optimal treatment, patient’s symptom experience and management, satisfaction with care and might predict prognosis, which has the potential to inform clinical decision-making based on the clinicians’ critical appraisal and interpretation of the available information [5, 34–36, 38–40, 44–61, 74].

Dirven et al. (2014) conducted a systematic review of PRO clinical trials in patient with brain tumours and demonstrated that HRQL can be used alongside overall and progression-free survival to inform clinical decision-making. One of the clinical trials included determined that the combination of concomitant and adjuvant temozolomide and radiotherapy has become standard care for newly diagnosed patients with glioblastoma [53]. This combination treatment led to significantly prolonged overall and progression-free survival, without negatively impacting HRQL in the long-term as measured with the EORTC QLQ-C30 questionnaire and Brain Cancer Module (BN-20) [53].

#### Shared decision-making

Shared decision-making is also important and four publications outlined the potential benefits of including PRO findings alongside other outcomes such as survival. This allowed patients and their clinicians to make an informed joint decision about treatment preferences and symptom management based on mutual understanding of treatment objectives and expectations [44, 58, 59, 73].

Sztankay et al. (2017) assessed HRQL during first-line chemotherapy with pametrexed and maintenance therapy (MT) among patients with advanced non-small cell lung cancer [73]. First-line chemotherapy for patients with advanced non-small cell lung cancer was shown to improve overall progression-free survival. However, as measured with the EORTC QLQ-C30 and EORTC QLQ-LC13, MT compared to first-line chemotherapy was associated with lower HRQL and improvements in nausea, vomiting, appetite loss, constipation and pain. This information presented alongside survival data, allowed patients and clinicians to make real-world informed joint decisions regarding treatment options.

#### Informed treatment consent

Consent for treatment refers to the authorisation given by a patient to receive a treatment, once the clinician presents a diagnosis, relevant treatment options and respective risks and benefits to the patient [97]. Two publications discussed the influence of PRO trial data on treatment consent [39, 49]. Parameswaran et al. (2008) presented a systematic review of two randomised controlled trials, 9 longitudinal studies and 11 cross-sectional studies. The authors determined that only 11 studies presented data that was capable of effectively informing patient consent. This statement was based on the assessment of the HRQL methodology of the studies, through the HRQL checklist by Efficace et al. (2003) [34]. For instance, as measured with the EORTC-QLQ-C30 and MOS-SF36, one of the eleven studies determined that surgery for oesophageal cancer patients has a detrimental impact on quality of life in the postoperative stage (e.g. anastomic leaks, sepsis and cardiac and pulmonary complications) and in some cases; quality of life among survival patients does not improve in the long term [39]. Therefore, communicating HRQL and clinical data to patients after the intervention could help inform patients about relevant information regarding recovery and outcomes before undergoing an intervention and inform the consent process.

## Abbreviations

CINALH+: Cumulative Index to Nursing and Allied Health Literature
CONSORT: Consolidated Standards of Reporting Trials
CONSORT-PRO Extension: Consolidated Standards of Reporting Trials-Patient Reported Outcomes Extension
EMA: European Medicine Agency
EMBASE: Excerpta Medica Database
EORTC QLQ-C30: European Organization for Research and Treatment - Core Quality of Life Questionnaire
EQ-5D: European Quality of Life Instrument - 5 Dimension
FDA: Food and Drug Administration
HADS: Hospital Anxiety and Depression Scale
HEIs: Higher Education Institutions
HMIC: Health Management Information Consortium
MEDLINE: Medical Literature Analysis and Retrieval System Online
MFSAF: Myelofibrosis Symptom Assessment Form
PCORI: Patient Centred Outcomes Research Institute
PRISMA: Preferred Reporting Items for Systematic Reviews and Meta-Analyses
PROs: Patient-reported outcomes
PROSPERO: The International Prospective Register of Systematic Reviews
PROTEUS: Patient-Reported Outcomes Tools: Engaging Users & Stakeholders
REF: Research Excellence Framework
SF-36: Short Form Health Survey 36-item
SISAQOL: The Setting International Standards in Analyzing Patient-Reported Outcomes and Quality of Life Endpoints Data
SPIRIT: Standard Protocol Items: Recommendations for Interventional Trial
SPIRIT-PRO Extension: Standard Protocol Items: Recommendations for Interventional Trial-Patient Reported Outcomes Extensions
UK: United Kingdom
VAS: Visual Analogue Scale
