# Supplementary material for: The impact of patient-reported outcome (PRO) data from clinical trials: a systematic review and critical analysis
Source: Health Qual Life Outcomes. 2019 Oct 16;17:156. doi: 10.1186/s12955-019-1220-z (PMC6796482; doi:10.1186/s12955-019-1220-z)
Supplement: Supplementary file 1 — Additional file 1. Search strategies [file 12955_2019_1220_MOESM1_ESM.docx]

**Appendix 1 - Search strategies**

Database: Ovid MEDLINE(R) <1946 to Dec Week 4 2018>

Search Strategy:

--------------------------------------------------------------------------------

1 (HRQL or HRQOL or QOL or quality of life or health index* or health indices or health profile* or health status or PROM* or PRO* or patient reported outcome* or self assessed outcome* or patient assessed outcome* or self report outcome* or health utility or patient report* outcome* or patient report* measure* patient report* assessment* or self report* outcome* or self* report* measure* or self* report* assessment* or self assess*).m_titl. (3561823)

2 (policy or health policy* or decision making or healthcare policy or policy making or policy initiative or reimbursement decision*).m_titl. (48587)

3 (clinical training or clinical practice or clinical guideline*).m_titl. (25046)

4 (healthcare training or healthcare practice or healthcare guideline*).m_titl. (84)

5 (labeling claims or labelling claims or promotional claims or drug approval).m_titl. (291)

6 2 or 3 or 4 or 5 (73779)

7 1 and 6 (9916)

8 (impact or influence or inform or role or implication* or integrati* or relationship*).m_titl. (1100499)

9 7 and 8 (1231)

***************************

Database: Embase <1974 to 2019 Jan 04>

Search Strategy:

1 (HRQL or HRQOL or QOL or quality of life or health index* or health indices or health profile* or health status or PROM* or PRO* or patient reported outcome* or self assessed outcome* or patient assessed outcome* or self report outcome* or health utility or patient report* outcome* or patient report* measure* patient report* assessment* or self report* outcome* or self* report* measure* or self* report* assessment* or self assess*).m_titl. (4680609)

2 (policy or health policy* or decision making or healthcare policy or policy making or policy initiative or reimbursement decision*).m_titl. (66221)

3 (clinical training or clinical practice or clinical guideline*).m_titl. (37567)

4 (healthcare training or healthcare practice or healthcare guideline*).m_titl. (116)

5 (labeling claims or labelling claims or promotional claims or drug approval).m_titl. (513)

6 2 or 3 or 4 or 5 (104122)

7 1 and 6 (14192)

8 (impact or influence or inform or role or implication* or integrati* or relationship*).m_titl. (1521903)

9 7 and 8 (1920)

***************************

Database: HMIC Health Management Information Consortium <1979 to January 2017>

Search Strategy:

--------------------------------------------------------------------------------

1 (HRQL or HRQOL or QOL or quality of life or health index* or health indices or health profile* or health status or PROM* or PRO* or patient reported outcome* or self assessed outcome* or patient assessed outcome* or self report outcome* or health utility or patient report* outcome* or patient report* measure* patient report* assessment* or self report* outcome* or self* report* measure* or self* report* assessment* or self assess*).m_titl. (51093)

2 (policy or health policy* or decision making or healthcare policy or policy making or policy initiative).m_titl. (7457)

3 (clinical training or clinical practice or clinical guideline*).m_titl. (1023)

4 (healthcare training or healthcare practice or healthcare guideline*).m_titl. (16)

5 (labeling claims or promotional claims).m_titl. (14)

6 2 or 3 or 4 or 5 (8485)

7 1 and 6 (1082)

8 (impact or influence or inform or role or implication* or integrati* or relationship*).m_titl. (17743)

9 7 and 8 (140)

***************************

Database: CINAHL+ <1979 to Dec 2018>

Search Strategy:

S1 TI HRQL or HRQOL or QOL or quality of life or health index* or health indices or health profile* or health status or PROM* or PRO* or patient reported outcome* or self assessed outcome* or patient assessed outcome* or self report outcome* or health utility or patient report* outcome* or patient report* measure* patient report* assessment* or self report* outcome* or self* report* measure* or self* report* assessment* or self assess* (662,953)

S2 TI policy or health policy* or decision making or healthcare policy or policy making or policy initiative (33,355)

S3 TI clinical training or clinical practice or clinical guideline* (17,158)

S4 TI healthcare training or healthcare practice or healthcare guideline* (956)

S5 TI labeling claims or promotional claims (20)

S6 S2 OR S3 OR S4 OR S5 (51,160)

S7 S1 AND S6 (8,086)
