# Supplementary material for: The impact of patient-reported outcome (PRO) data from clinical trials: a systematic review and critical analysis
Source: Health Qual Life Outcomes. 2019 Oct 16;17:156. doi: 10.1186/s12955-019-1220-z (PMC6796482; doi:10.1186/s12955-019-1220-z)
Supplement: Supplementary file 2 — Additional file 2. Systematic Review PRISMA Flow Diagram [file 12955_2019_1220_MOESM2_ESM.docx]

**Appendix 2 - Systematic Review PRISMA Flow Diagram**

Number of records identified (n=11,483) via:

- MEDLINE (Ovid) (n=1,231)
- EMBASE (n=1,920)
- HMIC (n=140)
- CINAHL+ (n=8,086)
- Google Scholar (n=100)
- Expert communication (n=6)


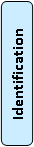


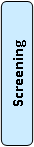

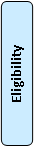

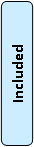


Number of papers excluded (n=16), reasons:

- PROs in routine care (n=10)
- PRO as an intervention (n=6)

Number of title/abstract records screened after 8,877 duplicates removed (n=2,606)

From: Moher D, Liberati A, Tetzlaff J, Altman DG, The PRISMA Group (2009). Preferred Reporting Items for Systematic Reviews and Meta Analyses: The PRISMA Statement.

Final number of full-text articles included **(n=39)**

Number of additional records included following hand-search of reference lists and citation searches (n=23)

Number of full-text records assessed for eligibility (n=32)

Number of records excluded (n=2,571), reasons:

- Out of scope (n=2,195)
- Routine clinical practice (n=68)
- PRO impact not discussed (n=20)
- Conference abstracts (n=288)
