# Supplementary material for: The impact of patient-reported outcome (PRO) data from clinical trials: a systematic review and critical analysis
Source: Health Qual Life Outcomes. 2019 Oct 16;17:156. doi: 10.1186/s12955-019-1220-z (PMC6796482; doi:10.1186/s12955-019-1220-z)
Supplement: Supplementary file 3 — Additional file 3. REF 2014 Impact case studies PRISMA flow diagram [file 12955_2019_1220_MOESM3_ESM.docx]

**Appendix 5 - REF 2014 Impact case studies PRISMA flow diagram**

**
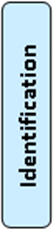
**

Number of records identified via REF 2014 database (n=209)

Number of duplicates (n=0)


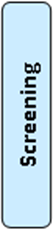
**
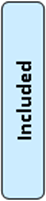

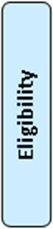
**

Number of full-text records assessed for eligibility (n=196)

Number of title/abstract records screened (n=209)

Number of papers excluded (n=128), reasons:

- Does not measure QoL within the clinical trial (n=111)
- The case study does not present a clinical trial (n=17)

Number of records excluded (n=13), reasons:

- The case study does not present a clinical trial (n=11)
- Animals (n=2)

Final number of full-text articles included (n=69)*

*Includes 3 duplicates (same case study submitted by two different institutions)
